# Metabolic syndrome in rheumatoid arthritis: case control study

**DOI:** 10.1186/1471-2474-14-147

**Published:** 2013-04-26

**Authors:** Samira Rostom, Mariam Mengat, Racha Lahlou, Asmaa Hari, Rachid Bahiri, Najia Hajjaj-Hassouni

**Affiliations:** 1 Department of Rheumatology, University Mohammed V Souissi. Faculty of Medicine and Pharmacy. El Ayachi hospital, University Hospital of Rabat-Sale, PO Box: 10000, Sale, Morocco

**Keywords:** Metabolic syndrome, Rheumatoid arthritis, Disease activity

## Abstract

**Background:**

Metabolic syndrome, a cluster of classical cardiovascular risk factors, including hypertension, obesity, glucose intolerance, and dyslipidemia is highly prevalent in patients with rheumatoid arthritis (RA). The aim of the study was to assess the frequency of metabolic syndrome (MS) in RA patients, and to evaluate the relationships between metabolic syndrome and RA.

**Methods:**

The study was conducted on 120 RA patients according to the 1987 revised American College of Rheumatology classification criteria, and 100 age and sex matched apparently healthy controls. The frequency of metabolic syndrome was assessed using six Metabolic Syndrome definitions (Joint Consensus 2009, National Cholesterol Education Programme 2004 and 2001, International Diabetes Federation, World Health Organisation and European Group for Study of Insulin Resistance). Logistic regression was used to identify independent predictors of metabolic Syndrome.

**Results:**

The frequency of metabolic syndrome varied from 18 to 48.6% in RA according to the definition used and was significantly higher than controls (for all definitions p<0.05). In multivariate analysis, higher ESR was independently associated with the presence of Met S (OR =1.36; CI: 1.18–2.12; *p* = 0.03). Glucocorticoid use, but not other disease modifying anti-rheumatic drugs (DMARDs), values remained significant independent predictors of the presence of metabolic syndrome in RA patients (OR = 1.45; CI: 1.12–2.14; *p* = 0.04).

**Conclusions:**

In summary, the frequency of metabolic syndrome in RA varies according to the definition used and was significantly higher compared to controls (for all definitions p<0.05). Higher systemic inflammatory marker, and glucocorticoids use were independent predictors associated with the presence of metabolic syndrome in patients with RA. These findings suggest that physicians should screen for metabolic syndrome in patients with RA to control its components and therefore reduce the risk of cardiovascular disease in these patients.

## Background

Rheumatoid arthritis (RA) is a chronic inflammatory disease associated with increased disability, morbidity and mortality [1,2]. CVD has turned out to be one of the most important causes of death in RA patients [3]. Therefore, the European League Against Rheumatism (EULAR) guidelines recommend that cardiovascular risk screening and management to be urgently done in patients with RA [4].

Metabolic syndrome and Inflammation are intimately linked. Inflammatory biomarkers are frequently elevated in people with Met S and conversely, the prevalence of Met S is higher in patients with chronic inflammatory rheumatic diseases [5].

Metabolic syndrome main components are dyslipidemia (elevated triglycerides and apolipoprotein B (apoB)-containing lipoproteins, and low high-density lipoproteins (HDL)), elevation of arterial blood pressure (BP) and dysregulated glucose homeostasis, while abdominal obesity and/or insulin resistance (IR) have gained increasing attention as the core manifestations of the syndrome [6]. Recently, other abnormalities such as prothrombotic states, non-alcoholic fatty liver disease and sleep apnea have been added to the entity of the syndrome, making its definition even more complex [6]. Besides the many components and clinical implications of metabolic syndrome, there are still no clearly defined diagnostic criteria. Several studies have examined the prevalence of Met S in RA subjects and whether it is increased compared to subjects without RA, but the results have been inconsistent, maybe due to the differences in metabolic syndrome definitions and in study populations [7,8]. The frequency of metabolic syndrome in RA patients is influenced not only by traditional factors such as race, age, and dietary habits, but also by disease-specific factors.

Morocco is one of the countries of North Africa with unmet need of sustainable healthcare delivery systems. North Africa is a region with challenging political, climatic and geographical conditions. Data from small studies in the region showed that RA is frequently diagnosed late and many patients present with active disease and severe disability. Despite this, only a small proportion of patients receive DMARDs particularly biologic DMARDS, and the scarcity of medical and social resources is a barrier to appropriate treatment in many countries [9-14]. Morocco had also participated in two international studies: QUEST RA study [15], which is a successful example of quantitative clinical measuring of RA as part of routine clinical care in a large number of centers across more than 30 countries including countries from Europe (France, Germany, Netherlands....), USA, Argentina, Brazil, Morocco, and United Arab Emirates. This study has suggested high disease activity in our country. The second study is COMORA study: comorbidities in RA (data not yet published), these two studies suggest a high disease activity in our country (300 patients from 10 centers of the country), the use of high-dose of corticoids, and a more frequent association with hyeprtension pressure, dyslipidemia, overweight and less frequent exposure to tobacco and alcohol. Solomon A et al. [16] have assessed the risk factor profiles for atherosclerotic cardiovascular disease in black and other Africans with established rheumatoid arthritis and found that proportions of individual metabolic syndrome components differed between black and other patients but their total numbers of metabolic risk factors and metabolic syndrome frequencies were similar. From these data from cross-sectional studies in Africa, we can assume that the activity profile and Severity of RA in Africa is quite different from Western countries. Furthermore, metabolic syndrome and activity are intimately linked. Hence, we aimed to assess the frequency of metabolic syndrome and its components and evaluate the supposed association with disease activity and severity of RA.

## Methods

### Sample

#### Patients

Our study included 120 consecutive patients with RA fulfilling the 1987 revised American College of Rheumatology classification criteria [17] over a period of 13 months (between May 2010 and June 2011) at the department of rheumatology of El Ayachi tertiary university hospital of Rabat-Sale from outpatient and inpatient services.

Patients with other inflammatory diseases, malignancies, diseases of the central nervous system, chronic kidney disease, chronic liver disease besides RA, were excluded from the study.

#### Healthy controls

One hundred age-, sex-, and race-matched apparently healthy volunteers’ women and men from urban and rural residences of the Rabat-Salé province in Morocco served as the control group in the present study. Healthy subjects were represented by families of patients (n=36), or families of medical or paramedical staff of the hospital (n=64).

Informed consent was obtained from all subjects. The study was approved by the Research and Ethical Review Board of the Avicenne University Hospital, Rabat, Morocco.

### Methods

#### Clinical assessment

Demographic characteristics, disease-specific variables (disease duration, duration of morning stiffness, the number of nocturnal awakenings, tender and swollen joint count…) drug use (all anti-rheumatic drugs, glucocorticoid use, cardiovascular drugs and analgesics among others), comorbid conditions, and family history of rheumatic and cardiovascular diseases were documented for each patient.

#### Disease activity and function

The Disease Activity Score including 28 joints (DAS28) was used, evaluating the number of swollen joints, number of tender joints, the patients’ global assessment of health measured on a visual analogic scale (VAS, range 0-100 mm), and erythrocyte sedimentation rate (ESR). A score of DAS28 between 2.6-3.2 indicates low disease activity, > 3.2- ≤ 5.1 moderate and > 5.1 high disease activity [18]. The patients also completed the validated Arabic translation of the Health Assessment Questionnaire (HAQ) [19], which served to evaluate functional disability; the HAQ score can range from 0 (no disability) to 3 (greatest possible disability). Pain and general health were measured by a visual analogic scale (VAS).

#### Body composition

Body mass index (BMI) was calculated from weight/ height2 (kg/m2). BMI values < 18.5 kg/m2 are considered underweight, between 18.5-24.9 as normal, 25-29.9 as overweight and values greater than 30 indicate obesity [20]. Waist circumference (WC) was measured to the nearest 0.5 cm midway between the iliac crest and the lower rib margin. According to the International Diabetes Federation (IDF) a waist circumference value less than 80 cm indicate low risk of type 2 diabetes, coronary heart disease or hypertension [21]. BP was measured by a mercury sphygmomanometer in the sitting position after five minutes of rest. Hypertension was defined by blood pressure ≥ 130 mmHg for systolic pressure or ≥ 85 mmHg diastolic pressure or current treatment for hypertension.

#### Biochemical measures

Biological tests were performed from venous blood samples obtained the morning after an overnight fast. Plasma fasting glucose (FG) levels were measured using the glucose oxydase method. C-reactive protein (CRP), ESR, total cholesterol, low-density lipoprotein (LDL) and high-density lipoprotein (HDL) were determined by standard laboratory methods. Concentrations of total cholesterol > 5.0 mmol/L, LDL ≥ 3.0 mmol/L, HDL < 1.3 mmol/L were considered pathologic [22]. A renal function assessment was performed by estimation of glomerular filtration rate according to the Modification of Diet in Renal Disease (MDRD) equation.

#### Metabolic syndrome

Metabolic syndrome is a cluster of classical cardiovascular risk factors (obesity, glucose intolerance, dyslipidaemia, and hypertension). It is thought to be associated with cardiovascular risk beyond the sum of its individual components [23], although this has recently been questioned.

##### Currently used criteria to define Met S

Despite the great interest in this subject, no consensus has been reached yet regarding the definition of Met S. Several groups have attempted to establish diagnostic criteria; the most widely used have been provided by many international organizations and expert groups, such as the World Health Organization (WHO) [24], the European Group for the study of Insulin Resistance (EGIR) [25], the National Cholesterol Education Program Adult Treatment Panel III (NCEP: ATPIII) [26], the International Diabetes Federation (IDF) [27] and the Joint Consensus (JC) [28]. They have attempted to incorporate all the different parameters used to define MetS (Table 1). In this study, the prevalence of Met S was assessed according to all existing definitions (JC, NCEP 2004, NCEP 2001, WHO, IDF, EGIR (Table 1) in order to establish the range of discrepancy between them. For further analysis of the predictors of metabolic syndrome, only the NCEP 2004 definition is presented, as it is the most widely used definition reported in the literature, thus allowing comparisons with other studies to be drawn [27].

**Table 1:** **A summary of the definitions of the metabolic syndrome**[20-27]

	**JC 2009**	**IDF (2005)**	**NCEP ATP III 2004**	**NCEP ATP III (2001)**	**EGIR 1999**	**WHO 1998**
**Number of criteria**	Three or more of:	And two or more of †:	Three or more of:	Three or more of:	And two or more of †:	And two or more of †:
**Obesity**	Population- and country-specific definitions *	WC ≥ 94 cm men, WC ≥ 80 cm women	WC ≥ 102 cm (men), WC ≥ 88 cm (women)	WC ≥ 102 cm (men), WC ≥ 88 cm (women)	WC ≥ 94 cm (men, WC ≥ 80 cm (women)	BMI > 30 and/or WHR > 0.9 (men), WHR > 0.85 (women)
**Blood Pressure mmhg**	≥ 130/85 or treatmen	≥130/≥85 or treatment	≥ 130/85 or treatment	≥ 130/85 or treatment	≥ 140/90	≥ 140/90
**Dyslipidaemia:HDL-C**	≥ 40 mg/dL (1.03 mol/L) in men, ≥ 50 mg/dL (1.29 mmol/L) in women, or treatment	≥ 40 mg/dL (1.03 mol/L) in men, ≥ 50 mg/dL (1.29 mmol/L) in women, or treatment	≥ 40 mg/dL (1.03 mol/L) in men, ≥ 50 mg/dL (1.29 mmol/L) in women, or treatment	≥ 40 mg/dL (1.03 mmol/L) in men or ≥ 50 mg/dL (1.29 mmol/L) in women	≥ 39 mg/dL (1.0 mmol/L) or treatment	≥ 35 mg/dL (0.9 mmol/L) in men or ≥ 39 mg/dL (≥ 1.0 mmol/L) in women
**Triglycerides**	≥150 mg/dL (1.7 mmol/L) or treatment	≥150 mg/dL (1.7 mmol/L) or treatment	≥150 mg/dL (1.7 mmol/L) or treatment	≥150 mg/dL (1.7 mmol/L)	≥150 mg/dL (1.7 mmol/L)	≥178 mg/dL (2.0 mmol/L) or treatment
**Glucose intolerance or fasting plasma glucose**	≥100 mg/dL (5.6 mmol/L) or T2D	≥100 mg/dL (5.6 mmol/L) or T2D	≥100 mg/dL (5.6 mmol/L) or T2D	≥110 mg/dL (6.1 mmol/L)	≥110 mg/dL (6.1 mmol/L)	≥110 mg/dL (6.1 mmol/l), *DM, IGT, IR*

* cut-off values differ according to ethnic origin , for Mediterranean and sub Saharian people, the threshold of obesity defined by WC corres pond to the IDF definition;

BMI = body mass index; JC= Joint Consensus; DM = diabetes mellitus; EGIR = European Group against Insulin Resistance; HDL-C = high-density lipoproteincholesterol; IDF = International Diabetes Federation; IGT = impaired glucose tolerance; IR = insulin resistance;NCEP ATPIII = National Cholesterol Education Programme Adult Treatment Panel; T2 D, type II diabetes mellitus; WC = waist circumference; WHO = World Health Organization; WHR = waist hip ratio.

†*For IDF 2005, EGIR 1999 and WHO 1998. The definition of metabolic syndrome focus on the presence of diabetes, glucose intolerance or insulin resistance together with the presence of at least two other components from a list of five components.*

### Statistical analysis

Statistical analysis was performed with SPSS 13, 0 statistical software packages. The statistical analysis of patient questionnaire data involved computation of means, medians, standard deviations, ranges for quantitative variables; and numbers and percentages for qualitative variables. Sample t test were used to compare quantitative data and the chi-square test for qualitative data. Multivariate logistic regression models were constructed and odds ratios (OR) and 95% confidence intervals (CI) were calculated to investigate the independence of the predictors of individual RA-related characteristics and Met S.

## Results

### Description of the RA patients

A summary of the socio-demographic and clinical characteristics of the patients is presented in Table 2. The mean age of the 120 patients examined was 49±12 years. Most of the patients were female 110 (91.7%). Patients had mean disease duration of 7.8 years, and had moderate disease activity (the mean DAS 28 score was 5±1.4), the mean HAQ score was 1.4±0.7. The majority of patients with RA were currently treated with disease-modifying anti-rheumatic drugs (DMARDs) (96%). The breakdown of DMARD usage was: 117 (96%) patients were taking methotrexate, 68 (56%) sulphasalazine, 21(18%) hydroxychloroquine and glucocorticoids (96%) with mean daily dose of 7.3±3 mg/j.

**Table 2:** Demographic and anthropometric characteristics of patients with RA and healthy controls

**Variables**	**RA patients (N=120)**	**Controls (N=100)**	***p*-value**
Age (years)^1^	49 ± 12	48.5 ± 13	0.78
female Sex^2^	110 (91.7 )	90 (90)	0.88
Menopause^2^	63(51.1)	61(61)	0.97
Body mass index^3^	28.1 (22.4 to 30.8)	24.5 (22.4 to 26.4)	0.23
Waist circumference^3^	91.4 (83.8 to 93.4)	80.4 (77.2 to 82.8)	0.001
Systolic blood pressure (mmHg)^3^	132.3 (126.1 to 130.5)	117.6 (115.5 to 119.7)	0.01
Diastolic blood pressure (mmHg)^3^	79.1 (77.9 to 80.3)	71.4 (70.3 to 74.5)	0.12
Total cholesterol, (mmol/l)^3^	5.4 (5.2 to 5.6)	5.2 (5.1 to 5.3)	0.32
HDL-cholesterol, (mmol/l)^3^	1.32 (1.25 to 1.37)	1.64 (1.60 to 1.70)	0.02
LDL-cholesterol, (mmol/l)^3^	3.3 (2.8 to 3.4)	2.4 (2.3 to 2.68)	0.04
Triglycerides, (mmol/l)^3^	2.09 (2.06 to 2.14)	1.97 (1.94 to 1.99)	0.23
Fasting plasma glucoce, (mmol/l)^3^	5.4 (5.3 to 5.5)	5.2 (5.1 to 5.4)	0.34

1: mean and standard deviation, 2: number and percentage, 3: median and quartiles.

### Frequency of metabolic syndrome in the study population according to the definition used and comparison with healthy controls

There was a great diversity in the reported prevalence rates according to the definition used (Table 3). The prevalence ranged from 18% to 48.6%. The most updated Joint Consensus 2009 criteria and most commonly used NCEP 2004 reporting a rate of 32.4%. For all definitions, metabolic syndrome had significantly lower frequency in control groups than in patients with RA (p < 0.05).

**Table 3:** Prevalence of metabolic syndrome according to definition used

**Definitions**	**JC 2009**	**IDF 2005**	**NCEP 2004**	**NCEP 2001**	**EGIR 1999**	**WHO 1998**
**Controls**	18	23	18	16	12	14
**Rheumatoid arthritis**	32.3*	48.6*	32.4*	24.6*	18*	20*

* For all definitions p<0.05.

### Associations of Metabolic syndrome in patients with RA

Characteristics of patients with RA who had and who did not have Met S are presented in Table 4. The Results presented were only for the NCEP 2004 criteria, but were very similar when we used other criteria, despite the difference in prevalence. In univariate analysis, patients in the groups with Met S were older (*p* = 0.03) and had a higher ESR (*P* = 0.02) and higher HAQ score (*p*= 0.03), than those without Met S. No significant differences were seen between the two groups according to disease activity and disease duration. TG, Fasting plasma glucoce and systolic blood pressure were significantly higher in the group with Met S (*P*= 0.04, *P*<0.001, *P* =0.03 respectively) compared with those who did not have the MetS. Methotrexate, Sulphasalazine, hydroxychloroquine use was not significantly associated with the presence of the Met S. Furthermore glucocorticoid use was associated with the presence of the Met S (*P*=0.003). The independence of each of these associations was tested, in a multivariate logistic regression model, an association between metabolic syndrome, ESR and glucocorticoids use persisted and remained a significant independent predictor of the presence of Met S in patients with RA (Table 5).

**Table 4:** Main characteristics of patients with rheumatoid arthritis included in this study according to the presence or absence of metabolic syndrome

**Characteristics**	**Total (n=120)**	**Mets absent (n=83)**	**Mets present (n=37)**	***P value***
Mean Age (years)^1^	49± 12	47±12	52±10	0.03
Female Sex n (%)^2^	110 (91.7 )	74(89.2)	36(97.3)	0.13
Menopause n(%)^2^	63(51.1)	38(45.8)	25(67.6)	0.02
RF positive n (%)^2^	93(76)	65(78.3)	28(75.7)	0.74
ACPA positive n (%)^2^	104(85)	73(88)	31(83)	0.53
Disease duration (yrs)^1^	7.8 ± 5.3	7.5±5.4	8.5± 4.9	0.31
CRP (mg/L)^1^	17(1à 112)	17±21	19±19	0.59
ESR (mm 1erhour)^1^	33± 23	24± 23	37± 24	0.04
DAS28^1^	5 ± 1.4	4.9±1.3	5.2± 1.5	0.32
Global pain (VAS : 0-100 mm)^1^	54 ± 21	57±17	53±20	0.36
HAQ^1^	1.4± 0.7	1.1±0.7	1.5±0.7	0.04
Methotrexate n (%)^2^	117(96)	80(96)	37(100)	0.24
glucocorticoids mean daily dose (mg/j)^1^	7.3±3.05	7.5±2.3	8±2.4	0.44

^1^: Mean ± standard deviation, ^2^: number (percentage). ACPA = anti-cyclic citrullinated peptide; BP = blood pressure; CRP = C-reactive protein; DAS =Disease Activity Score; ESR = erythrocyte sedimentation rate; HAQ = Health Assessment Questionnaire; HDL =high-density lipoprotein; MetS = metabolic syndrome; RA = rheumatoid arthritis; RF = rheumatoid facto; BMI= Body mass index; VAS= visual analogic scale.

**Table 5:** Odds ratios for having the metabolic syndrome in patients with RA

	**Univariate analysis**			**Multivariate analysis**		
	**OR**	**IC95%**	***P***	**OR**	**95% CI**	***P***
**Age (years)**	1.33	1.19-2.35	0.03	1.13	1.18-1.76	0.07
**Disease duration (years)**	1.03	0.96-1.11	0.32			
**DAS28**	1.16	0.87-1.55	0.28			
**HAQ**	1.45	1.12-2.5	0.03	1. 13	0.90-2.13	0.16
**MTX**	0.01	0.03-1.7	0.76			
**Glucocorticoids use**	1.93	1.34-3.92	0.003	1.45	1.12-2.14	0.04
**CRP**	1.01	0.88-1.02	0.60			
**ESR**	2.21	1.99-2.62	0.002	1.36	1.18-2.12	0.03

DAS =Disease Activity Score; ESR = erythrocyte sedimentation rate; HAQ = Health Assessment Questionnaire; CRP = C-reactive protein; MTX =methotrexate. The factors included in the multivariate analysis were age, HAQ, glucorticoids use, and ESR.

## Discussion

This case-control study suggested that RA is associated with increased frequency of metabolic syndrome compared to control groups, but its frequency depends on the definition used. Higher systemic inflammatory markers (ESR) and glucocorticoid use were independent predictors associated with the presence of metabolic syndrome in patients with RA.

The metabolic syndrome describes a constellation of major risk factors for cardiovascular diseases (CVDs) such as atherogenic dyslipidemia, obesity, hypertension and diabetes. These associated risk factors have been previously called syndrome X2 or the insulin resistance syndrome [29].

The frequency of metabolic syndrome has varied markedly between different studies [30], most likely because of the lack of accepted criteria for the definition of metabolic syndrome. Recently, Karvounaris *et al* found a frequency of metabolic syndrome (defined according to the NCEP ATP III criteria) in RA patients (40%), comparable with their control population [29]. In South Asia, Dodani and al found that the frequency of metabolic syndrome was 13.3% and 40%, respectively, according to WHO and NCEP ATP III criteria [31]. Another study showed that metabolic syndrome was significantly more prevalent in American patients with long-standing RA (42% – WHO and NCEP/ATPIII criteria) as well as in early RA patients (31% and 30% – WHO and NCEPIII criteria respectively) than in controls (11% and 22% – WHO and NCEP/ATPIII criteria, respectively) [32]. Allowing comparison of our results with those of other studies in RA and other conditions, In the literature, the frequency of metabolic syndrome varied considerably, even using the same criteria; for example, using the NCEP 2001, the frequency ranged from 17% in Mexican [33], 19% in South African [7], 19.9% in Dutch [34], 38.3% in English [35], to 41.5% in Swedish [36], 42% in American [32], and 44% in Greek [29] patients with RA and 24.6% in our study. Such diversity can be explained by differences in the baseline characteristics and disease characteristics [37].

The factors found in this study to be associated independently with the metabolic syndrome in RA, irrespective of the definition used, included higher systemic inflammatory markers, and glucocorticoid usage. In our study, we didn’t find an association between older age and metabolic syndrome contrary to others studies [15,16]. The association with older age is not surprising, because in the general population, metabolic syndrome has been shown to affect primarily older subjects, as a consequence of age-related modification of some of its components [38]. The association between higher ESR and the presence of metabolic syndrome in patients with RA in our study was also previously reported [38]. These findings further support the role of chronic inflammation in insulin resistance development [39,40]. However, ESR can be affected by a number of comorbid conditions (renal disease) as well as age. Furthermore, metabolic syndrome is an inflammatory condition, and thus ESR may be elevated to some degree by the presence of metabolic syndrome. Moreover, the cross-sectional design of our study limit the ability to describe causal relationships to the associations detected.

Another finding from this study was the association between glucocorticoids use and the presence of metabolic syndrome. This relationship may be explained by the effects of glucocorticoids on different components of the lipid profile. To the contrary, the study among Mediterranean RA patients found that metabolic syndrome in RA is independent of gender and body mass index (BMI) [29,32]. Current corticosteroid use does not increase the risk of metabolic syndrome [35,41] while it may even reduce its prevalence [36]. On the other hand, the risk of having moderate to severe RA is higher in patients with metabolic syndrome than in those without the syndrome [29]. Furthermore, another study showed that the use of glucocorticoids is not associated with the presence of metabolic syndrome [41]. Dessein and coworkers found that corticosteroid use was not associated with dyslipidemia in RA patients [42] but was associated with insulin resistance. Therefore, the lipoprotein effects of glucocorticoids are unlikely to be the sole cause (if any) of the impact of these drugs on cardiovascular risk. Indeed, the analysis of lipid levels in the COBRA (“Combination therapy in rheumatoid arthritis”) study found that combination therapy with glucocorticoids, methotrexate, and sulfasalazine was associated with a more rapid and favorable impact on the atherogenic index (total/HDL cholesterol ratio) in RA [42]. In the literature, Glucocorticoid use is associated with adverse lipid profiles in the general population, and its long-term use is a risk factor for CVD [43]. However, the relationship between glucocorticoid use and cardiovascular risk in patients with RA is complicated by the fact that these drugs tend to be used more often in patients with severe or intractable disease; therefore, it is difficult to determine whether the disease or the treatment increase the risk [44].

The limitations of the current study should be addressed. The cross-sectional design and small size of our study limit the ability to describe causal relationships to the associations detected. Furthermore; the small sample size of this study, and recruitment from a single center, which is a tertiary care center with recruitment of the most active and severe disease expose to bias selection and the risk of surestmation of the real frequency of metabolic syndrome. The international COMORA study (comorbidities in RA); in which Morocco has participated with 300 patients across 10 centers in the country (private and public structures) will be interesting, it can given the frequency of metabolic syndrome and its components and compared these data through countries. Moreover, the Moroccan ESPOIR study (Etude et Suivi des POlyarthrites rhumatoïdes et arthrites Indifférenciées Récentes), a prospective multicenter cohort study of 10 years of follow-up will provide some information on the epidemiological profile of the RA in Morocco. Further prospective studies should prove valuable in determining these causal relationships.

## Conclusion

In summary, this study shows that rheum arthritis has been associated with increased prevalence of metabolic syndrome compared to control groups. A higher systemic inflammatory marker and glucocorticoid use were independent predictors associated with the presence of metabolic syndrome in patients with RA. These findings suggest that clinicians should screen for metabolic syndrome in patients with RA to control its components and, therefore, reduce the risk of cardiovascular disease in these patients

## Competing interests

The authors declare that they have no competing interests.

## Authors’ contributions

RS: have made substantial contributions to conception and design, acquisition of data, and analysis and interpretation of data. Participated in the sequence alignment and drafted the manuscript. MM: have made substantial contributions to acquisition of data, analysis of data. LR: have made substantial contributions to acquisition of data. HA: have made substantial contributions to acquisition of data. BR: have given final approval of the version to be published. HHN: have been involved in drafting the manuscript or revising it critically for important intellectual content and given final approval of the version to be published. All authors read and approved the final manuscript.

## Pre-publication history

The pre-publication history for this paper can be accessed here:

http://www.biomedcentral.com/1471-2474/14/147/prepub
